# Gait Impairment and Alzheimer's Disease Pathology: A Narrative Review on Mechanistic Links

**DOI:** 10.1111/ggi.70675

**Published:** 2026-08-02

**Authors:** Ryota Sakurai, Manuel Montero‐Odasso

**Affiliations:** ^1^ Research Team for Social Participation and Healthy Aging, Tokyo Metropolitan Institute for Geriatrics and Gerontology Tokyo Japan; ^2^ Gait & Brain Lab Lawson Research Institute, Parkwood Institute London Ontario Canada; ^3^ Division of Geriatric Medicine, Department of Medicine, Schulich School of Medicine and Dentistry Western University London Ontario Canada; ^4^ Department of Epidemiology and Biostatistics, Schulich School of Medicine and Dentistry Western University London Ontario Canada

**Keywords:** Alzheimer's disease, amyloid‐β, gait, neurodegeneration, tau

## Abstract

In older adults, gait has emerged as an important indicator of overall health and a strong predictor of adverse outcomes, including dementia. This association has been corroborated by findings from Alzheimer's disease (AD) research. In AD, amyloid‐β brain accumulation is succeeded by tau pathology and neurodegeneration, commencing within the medial temporal lobe. Older adults exhibiting slower gait speed or reduced gait adaptability display greater amyloid and tau brain deposition, as well as more pronounced hippocampal atrophy, suggesting that gait impairment may serve as an early clinical marker of AD‐related neurodegeneration. Despite accumulating evidence linking gait impairment to AD‐related pathology, the underlying mechanisms remain inadequately understood. Traditional explanations have focused on shared neural substrates, including frontal–subcortical and motor control networks, which decline with aging and result in parallel deterioration of gait and executive function. Although this framework aligns with cognitive reserve theory, it fails to fully explain the potential pathways linking gait disturbances to AD‐related and mixed brain pathology. In this review, we explore interacting mechanisms suggesting that gait impairment and AD‐related changes may arise from common vulnerabilities and mutually reinforcing processes. By synthesizing the current evidence, we aim to advance the understanding of gait decline as a prodromal symptom of dementia, advocate for early screening of gait performance, and highlight the importance of maintaining gait function across the lifespan as part of healthy aging strategies that may help delay the onset of dementia. The conclusion underscores a life‐course perspective on health, rather than one that focuses solely on functional decline in old age.

## Graphical abstract

Gait impairment and AD‐related pathology may be linked through shared risks, preclinical AD changes, feedback effects of persistent gait dysfunction, and life‐course susceptibility, underscoring the importance of maintaining brain–body health across the lifespan.

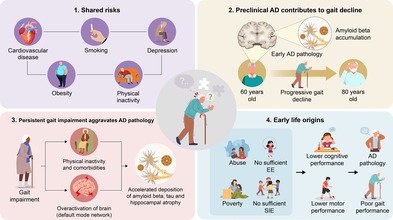

## 1 Established Association Between Gait and Cognition

Gait is being increasingly recognized as a crucial marker of overall health and a robust predictor of adverse outcomes in older adults, including falls, frailty, cognitive impairment, and dementia [1, 2, 3]. Over the past two decades, accumulating evidence has shown that age‐related gait impairments, particularly slowing gait speed and increased gait variability, are associated with a higher risk of dementia onset, even in the absence of overt neurological disease [4]. Motor impairments, such as gait slowing, tremor, and parkinsonian features, may precede cognitive decline; among these, lower‐limb motor performance, especially reduced gait speed and increased gait variability, shows the strongest association with impaired cognition [5]. For example, older adults with gait abnormalities are reported to have a 1.2–2.3‐fold higher risk of developing dementia, primarily Alzheimer's disease (AD), over follow‐up periods of 5–12 years [6, 7, 8, 9, 10].

AD is the most prevalent cause of dementia, and it is hypothesized that the cognitive decline is primarily driven by amyloid‐β (Aβ) brain accumulation, followed by tau pathology and subsequent neurodegeneration in the medial temporal lobe, including the hippocampus and entorhinal cortex. Older adults displaying diminished gait performance, such as slower gait speed, exhibit greater Aβ and tau deposition, with more pronounced brain atrophy, mainly in the hippocampus [11, 12, 13]. These findings reinforce the plausibility of using gait as an early marker of dementia risk.

Nevertheless, significant gaps remain. Evidence linking gait performance with AD‐related pathology is fragmented, and the mechanisms underlying this association have not been fully elucidated. Therefore, herein, we reviewed the literature studying potential pathways linking gait impairment with AD pathology, with particular emphasis on the ATN (Aβ, tau, and neurodegeneration) framework [14], a biomarker‐based classification system for diagnosing and staging AD, to structure our discussion of AD‐related changes (details of the search strategy are provided in Supplementary File 1). First, we examine existing studies that report associations between gait performance and emerging AD pathology, stratifying the findings based on cognitive status. Older adults with and without mild cognitive impairment (MCI), a prodromal AD state, are generally evaluated to determine whether dementia risk modifies the relationship. We also searched on gait performance under single‐ and dual‐task conditions. Dual‐task paradigms on gait, assessing walking performance while performing a cognitively demanding task, are useful for evaluating cognitive–motor interactions and for screening early cognitive decline [3, 15]. Although emerging evidence suggests that low performance in dual‐task gait is associated with increased dementia incidence [16], the role of AD pathology remains poorly understood.

Of note, gait impairments are not unique to AD and occur across a range of neurodegenerative conditions associated with dementia. For example, Parkinson's disease and dementia with Lewy bodies are characterized by shuffling and stooped gait, gait freezing, and postural instability [17, 18], whereas vascular dementia often presents with gait variability, lower‐body parkinsonism, and hemiplegic gait [19]. In contrast, gait impairments in early AD tend to be more subtle, typically manifesting as slowed gait speed or impaired dual‐task performance, rather than overt motor symptoms [20]. Furthermore, emerging evidence suggests that clinical AD often involves mixed and microvascular pathologies, which may further complicate the interpretation of associations between gait impairment and AD‐related pathology [21]. These distinctions indicate that gait impairment reflects a combination of disease‐specific features and shared neurodegenerative processes. Therefore, considering the broader context of mixed pathologies in neurodegeneration is essential when interpreting associations between gait performance and AD‐related pathology.

Traditionally, the link between gait and cognition has been attributed to shared neural substrates, particularly frontal–subcortical circuits that are crucial for controlling gait and navigation and for the performance of the cognitive domains of executive functions and memory [3, 22, 23]. As these networks deteriorate with aging, executive functions, and motor control may decline in parallel. This overlap is evident in dual‐task paradigms, where cognitive load exacerbates gait disturbances, suggesting a shared underlying deficit [15]. Although this view aligns with the cognitive reserve hypothesis, which describes the brain's ability to compensate for damage, it does not fully explain why gait impairment often appears alongside AD‐related and mixed pathology. In this review, we conceptualize gait impairment as a potential early manifestation of AD‐related alterations, rather than a causal contributor, while acknowledging that gait and cognitive impairments likely emerge through overlapping and partially distinct mechanisms. Through this review, we sought to advance the understanding of gait impairment as a prodromal feature of dementia and to highlight its potential utility as a non‐cognitive marker for dementia screening while emphasizing the importance of preserving gait function as part of multidomain healthy aging strategies that may help delay dementia onset.

## 2 Gait Performance and AD‐Related Pathology

### 2.1 Simple Gait Performance Among Cognitively Normal Older Adults

In cognitively normal older adults, impairment in a simple gait performance (single‐task walking) is associated with AD‐related pathology. Studies consistently indicate that a higher Aβ brain burden, measured using positron emission tomography (PET), correlates with reduced gait speed [11, 24, 25, 26] and increased gait variability, a marker of poor gait stability [27]. Biomarker studies using plasma indices further validate these associations [12, 28]. For example, elevated levels of phosphorylated‐tau (p‐tau) 181 and neurofilament light chain (NfL) in plasma have been linked to slower gait speed, although no associations have been identified with the Aβ42/Aβ40 ratio, p‐tau217, or glial fibrillary acidic protein (GFAP) level [12]. Another study reported significant associations of multiple gait parameters with GFAP, NfL, and p‐tau181 levels [29]. Neuroimaging findings are consistent with these results, with hippocampal atrophy being associated with slower gait, shorter stride length, and increased gait variability [13, 30, 31, 32, 33, 34]. Conversely, one study reported that greater hippocampal volume correlates with greater stride time variability, suggesting the possibility of a compensatory mechanism involved in maintaining physiological gait control [35]. Diffusion tensor imaging studies have shown that lower gray matter integrity, specifically in the hippocampus and anterior cingulate, is associated with higher step variability [36]. Moreover, fluorodeoxyglucose‐PET studies have demonstrated that reduced glucose metabolism in the posterior cingulate cortex, an early site of AD‐related hypometabolism, correlates with slower fast‐paced gait [23, 37]. Together, these findings suggest that gait performance may capture early or subtle brain changes that are not apparent in global cognitive testing, although such changes likely reflect a combination of AD‐related and non‐AD neurodegenerative processes [38].

### 2.2 Simple Gait Performance Among Older Adults With MCI

Gait abnormalities among older adults with MCI are also related to AD biomarkers, although the evidence is limited. Increased tau accumulation detected using PET has been associated with greater step velocity variability, whereas no significant associations with Aβ accumulation or white matter hyperintensities have been identified [39]. Another study reported that shorter stride length is strongly associated with elevated p‐tau181 plasma levels but not with Aβ42/Aβ40 plasma levels [40]. Structural neuroimaging revealed that, in individuals with MCI, gait speed and stride length correlate with parahippocampal gyrus volume, whereas in cognitively normal controls, they correlate with frontal region volumes [41]. Poor performance in the Timed Up and Go (TUG) test has been linked to smaller hippocampal volume in older adults with and without MCI [42]. Furthermore, enlarged ventricular volume, reflecting global brain atrophy, has been associated with slower gait and greater gait abnormalities, suggesting a role for widespread neurodegenerative processes that may contribute to AD onset and mixed pathology [43, 44].

### 2.3 Dual‐Task Gait Performance Among Cognitively Normal Older Adults

Although limited in number, some studies suggest that dual‐task gait performance in cognitively normal older adults is moderately associated with Aβ brain deposition. In studies employing working‐memory, motor‐sequencing, and phone‐dialing tasks, the degree of gait slowing during dual‐task conditions (i.e., dual‐task cost) has been found to be significantly correlated with Aβ brain burden measured using Pittsburgh Compound B (PiB) imaging, with correlation coefficients ranging 0.39–0.48; in contrast, no such relationship has been observed for single‐task gait speed [45]. Similarly, a study using florbetapir‐PET demonstrated that poorer dual‐task performance on the TUG test predicts greater cerebral Aβ accumulation [46]. In addition, decreased trunk stability during dual‐task walking has been shown to be significantly associated with brain atrophy in older adults [47]. Together, these studies suggest that early changes in gait performance during cognitively demanding tasks may serve as an early indicator of AD‐related and mixed pathology. However, when comparing cognitively normal participants and participants with MCI, one study found no association between AD biomarkers in the cerebrospinal fluid (CSF) and dual‐task gait performance among cognitively normal older adults [48].

### 2.4 Dual‐Task Gait Performance Among Older Adults With MCI

Evidence from individuals with MCI suggests that dual‐task gait is more strongly associated with tau pathology than with Aβ brain burden. A study using principal component analysis of tri‐axial accelerometer data revealed that rhythm‐related gait parameters (mean stance, stride, and swing times, and cadence) under dual‐task conditions are associated with CSF total tau level but not Aβ level [49]. Another study indicated that production of fewer correct words during the dual‐task TUG test is correlated with higher CSF total and p‐tau levels, again showing no association with Aβ level [50]. Notably, both studies included participants with a range of cognitive symptoms and did not focus exclusively on MCI, limiting the generalizability of these findings. However, a separate study examining CSF Aβ42/40 and CSF p‐tau levels reported that the significant association between poorer dual‐task TUG test performance and CSF p‐tau level was present only in older adults with MCI, and not in cognitively healthy older adults [48]. Regarding brain structural changes, smaller entorhinal cortex volume has been linked to poorer dual‐task gait performance but not single‐task gait performance in MCI [51]. Hippocampal atrophy has also been associated with lower dual‐task performance in another study, although not all participants had MCI [52]. Consistent with these findings, greater gray matter volume in the cingulate cortex has been related to better dual‐task gait performance, suggesting the involvement of attention and executive networks [53]. Moreover, a follow‐up study demonstrated that reduced gray matter volume in the right anterior and middle cingulate cortices mediates the relationship between poor dual‐task gait and incident dementia among individuals with MCI [54].

### 2.5 Interpretation of Epidemiological Findings of Gait and AD Pathology

Although findings vary across studies, impaired simple‐ and dual‐task gait performance have both been associated with AD‐related pathology. However, the strength and consistency of this evidence differ by cognitive status. Associations have been more consistently reported in cognitively normal older adults and in mixed samples including individuals with MCI, whereas studies restricted to MCI populations remain relatively few and suggest stronger links with tau or neurodegeneration than with Aβ. A possible explanation is that the pathological spectrum in individuals with MCI is relatively narrow, resulting in limited variability in gait performance and AD biomarkers, which may obscure detectable associations. In contrast, although not the primary focus of this review, studies including cognitively normal participants and participants with MCI tend to report stronger associations between gait and AD‐related pathology. In addition, the heterogeneity and inconsistency of the reported findings may partly reflect the inclusion of Aβ‐negative individuals who exhibit tau pathology or neurodegeneration, as well as the frequent coexistence of other pathologies, such as Lewy body disease and microvascular changes. Therefore, epidemiological associations between gait and AD‐related pathology should be interpreted with caution, given the heterogeneity of underlying disease processes and potential contribution of mixed pathologies.

Although some studies suggest significance [45, 51], we did not find consistent evidence that dual‐task gait performance is more strongly associated with AD pathology, compared with single‐task gait performance. Dual‐task gait performance refers to performing an attention‐demanding task while walking [55]. The underlying hypothesis is that two simultaneously performed tasks interfere with and compete for brain resources [56]. Dual‐task gait has been proposed and used as a tool to assess the impact of cognitive deficits on gross motor performance, gait stability and navigation, and falls risk [56]. Consequently, dual‐task gait assessment can act as a “brain stress test” that detects impeding cognitive decline. Gait modifications during dual‐tasking (also known as dual‐task costs), such as slowed gait, are interpreted as reflecting the increased cognitive demand on cortical attention processes while walking. A previous study demonstrated that slow single‐task gait speed is not associated with progression from MCI to dementia, whereas high dual‐task cost (i.e., poor dual‐task gait performance) is associated with dementia progression, suggesting the superiority of dual‐task gait over simple gait performance [16]. However, given the lack of a consistently strong association between dual‐task gait performance and AD‐related pathology, gait impairment is better framed as an early functional correlate of underlying pathological changes, rather than a direct preclinical marker. Dual‐task gait impairment likely reflects an early disruption in cognitive–motor integration, whereas single‐task gait impairment may capture a broader, less specific motor decline. In line with this view, a recent study suggested that dual‐task gait may better capture Aβ‐related effects, particularly through its interaction with cognitive function, despite single‐ and dual‐task gait being associated with Aβ deposition [57]. Further research is needed to determine whether dual‐task gait performance provides meaningful insight into dementia onset.

If AD‐related changes are indeed linked to gait function, then from a temporal perspective, Aβ accumulation would be expected to show the strongest association. Nevertheless, several studies have reported selective associations with tau or NfL but not with Aβ. This suggests that the relationship between gait and ATN biomarkers may not align perfectly with the clinical progression of AD. Instead, it may reflect the interactions among the ATN components or disease‐specific features of each pathology; for example, motor impairments associated with tauopathy or brain atrophy due to cerebrovascular disease. Together, these findings suggest that gait impairment should not be interpreted as a direct marker of AD‐specific pathology but as an early functional indicator of brain vulnerability associated with AD‐related and mixed neurodegenerative processes. The independent effects of Aβ and tau pathology are discussed further in Section 3.2.

## 3 Mechanisms Linking Gait Performance and AD‐Related Pathology

**FIGURE 1:**
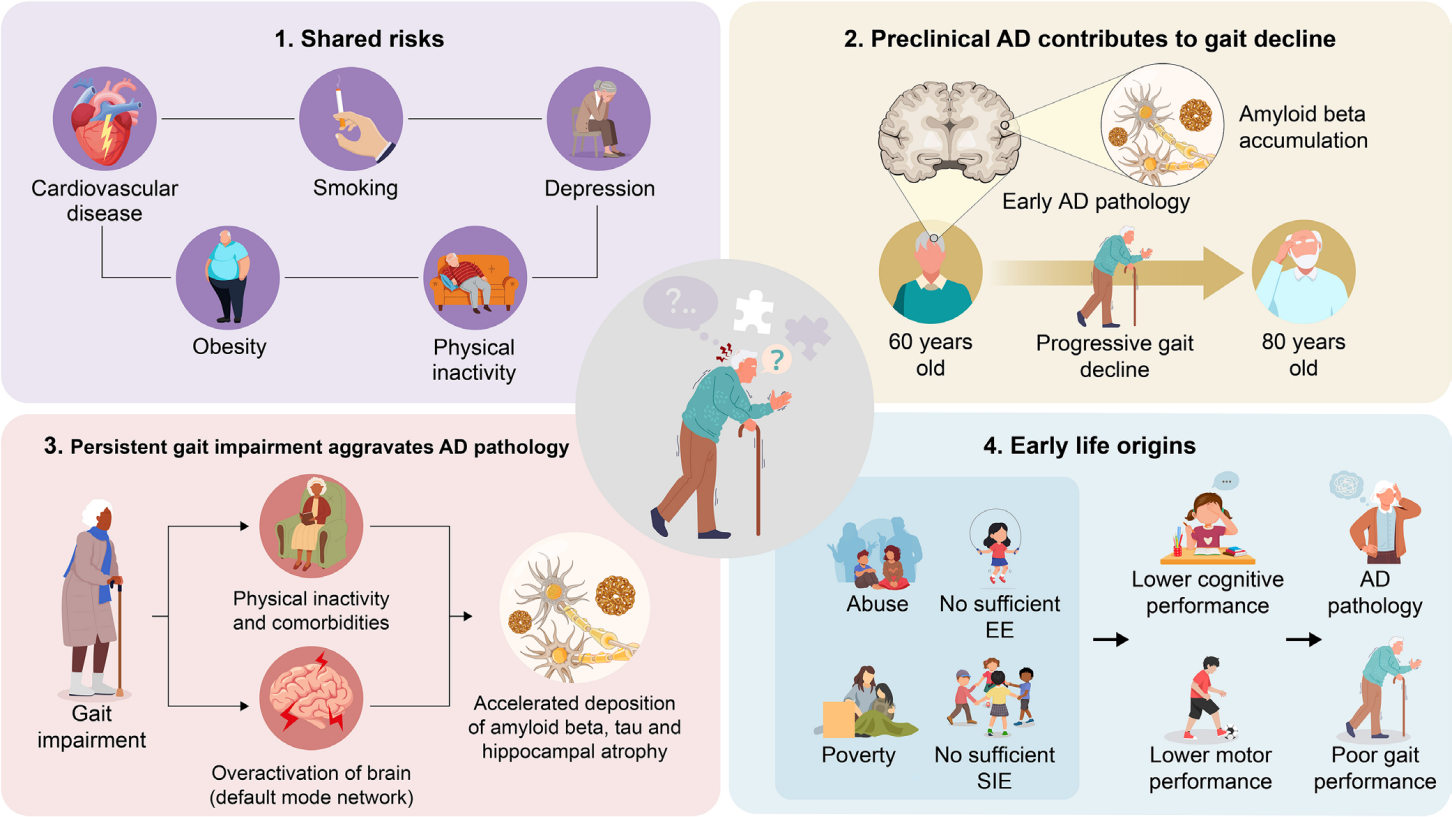
Conceptual diagram of hypothetical mechanisms underlying the relationship between gait impairment and the occurrence of Alzheimer's disease (AD)‐related pathology. Although this review highlights cardiovascular disease, obesity, smoking, physical inactivity, and depression as shared modifiable risk factors for gait impairment and AD‐related pathology, other potentially modifiable factors, such as metabolic disorders, vision loss, hearing loss, and traumatic brain injury, may also represent potential shared risk factors and are summarized in Supplementary File 2. EE, exercise experience; SIE, social interaction experience.

This section delineates and proposes mechanisms to explain the association between gait impairment and AD‐related pathology, depicted in Figure 1. These mechanisms should not be interpreted as AD‐specific processes; instead, they represent broader biological and systemic pathways that contribute to gait dysfunction and brain vulnerability, including mixed neurodegenerative pathologies. Furthermore, these mechanisms are not mutually exclusive; they may overlap and interact dynamically in older adults, thereby increasing the likelihood that gait disturbances co‐occur with AD‐related neurodegeneration. Although some risk factors are shared, the causal mechanisms underlying gait impairment and the emergence of AD pathology are not necessarily identical.

### 3.1 Common Risk Factors Influencing Gait and AD‐Related Pathology

**FIGURE 2:**
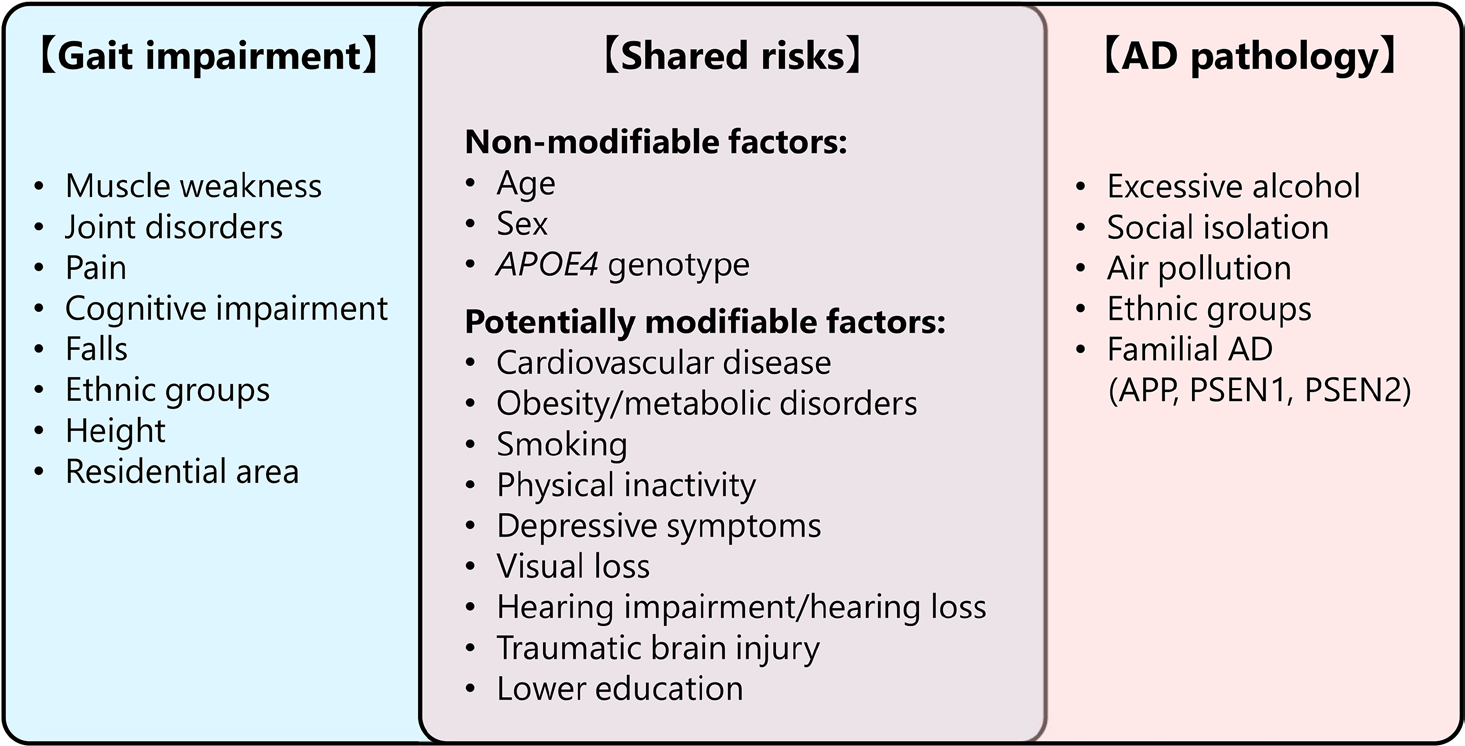
Shared and distinct risks of gait impairment and Alzheimer's disease (AD)‐related pathology. The classification is based on systematic studies assessing risks for gait impairment and dementia. A risk factor with unknown mechanisms (e.g., ethnic groups) is not classified as shared risks in this study, even though they are associated with gait impairment and AD pathology. Although cognitive impairment is associated with AD pathology, it largely reflects the consequences of cardiovascular disease or AD pathology and is treated here as an individual risk factor rather than a shared risk factor. Although social isolation has been reported to be associated with both gait impairment and AD pathology, we treated it here as an individual factor because its effects may be largely mediated by intervening factors such as physical inactivity. Additional modifiable and non‐modifiable factors associated with gait impairment and AD‐related pathology are summarized in Supplementary File 2 as they are not discussed in detail in the main text.

Large population‐based studies have shown that variation in gait speed or the onset of slow gait is significantly associated with modifiable factors, including cardiovascular disease, depressive symptoms, physical inactivity, muscle weakness, smoking, obesity, pain, cognitive impairment, and falls [58, 59, 60]. Among these, this review highlights cardiovascular disease, obesity, smoking, physical inactivity, and depression as shared modifiable risk factors for gait impairment and AD‐related pathology, given their overlap with established dementia risk (Figure 2) [61].

Although systematic studies are limited, metabolic disorders, vision loss, hearing loss, and traumatic brain injury which are well‐known risk factors for dementia, are also associated with gait impairment, suggesting that they may be potential shared risk factors (Supplementary File 2). Well‐established non‐modifiable dementia risk factors, including age, sex, and genetics (i.e., the apolipoprotein ε4 allele genotype, *APOE* ε4), are likewise associated with gait impairment and are considered here as non‐modifiable shared risk factors (Supplementary File 2).

These factors are not specific to AD and are more appropriately interpreted as common upstream contributors to gait dysfunction and broader brain vulnerability, including mixed pathologies. Given their interrelated nature, these factors are better understood as a constellation of interacting influences, rather than as simple one‐to‐one relationships. Through these shared risks, impaired gait performance may emerge as an early, non‐cognitive indicator of vulnerability to AD‐related pathological changes.

#### 3.1.1 Cardiovascular Disease

Hypertension and other cardiovascular conditions are among the established modifiable risk factors for dementia. Elevated blood pressure contributes to AD by damaging cerebral vasculature, impairing perfusion, and disrupting the blood–brain barrier, leading to microbleeds, ischemia, and the accumulation of toxic proteins, such as Aβ [62, 63]. Furthermore, atherosclerosis is associated with hypoxia, inflammation, oxidative stress, and the accumulation of advanced glycation end products [64]. These vascular injuries can accelerate the deposition and/or reduce clearance of amyloid in the brain, promote microglial activation, and ultimately exacerbate neurodegeneration and brain volume loss [62, 63].

The same vascular pathologies are associated with gait disturbances [65]. Atherosclerosis, white matter hyperintensities, and lacunar infarcts cause chronic cerebral hypoperfusion and impair neural connectivity in motor and executive control circuits [66, 67]. Such disruptions manifest clinically as slower gait, reduced stride length, and impaired balance, even in cognitively normal older adults. Notably, hypertension and dyslipidemia increase the risk of concurrent declines in gait speed and cognition, showing the highest risk of progression to dementia [68].

Concomitant cerebrovascular pathology is a common finding in brain autopsy studies of older adults with neurodegenerative conditions, including AD, and is also often observed in Parkinson's disease and frontotemporal dementia [69, 70, 71]. The coexistence of cerebrovascular disease in individuals with neurodegenerative disorders has been associated with accelerated neurodegenerative changes, suggesting a synergistic effect on overall brain vulnerability [72]. In addition, vascular risk burden is highly prevalent across neurocognitive syndromes, including MCI, AD, and frontotemporal and vascular dementia, and is known to impair white matter integrity, which plays a critical role in cognitive and motor function [73]. Given the close interrelationships among cerebrovascular pathology, AD‐related pathological changes, and gait performance, vascular mechanisms may represent one of the most influential and biologically plausible underlying factors linking gait impairment and cognitive decline. In this context, cerebrovascular disease can be considered a key background factor shaping the association between gait dysfunction and AD‐related pathology.

#### 3.1.2 Obesity

Obesity contributes to impaired insulin signaling, altered synaptic plasticity, chronic inflammation, and oxidative stress, all of which disrupt Aβ clearance and promote its aggregation, thereby increasing brain damage and the risk of AD [74, 75]. Evidence from animal models indicates that obesity and related metabolic disturbances play a significant role in AD pathophysiology [76, 77, 78]. Although the mechanism is plausible, findings from human studies remain inconsistent regarding the association between obesity and AD pathology [79, 80, 81].

Obesity is also linked to gait abnormalities, including slower gait speed, greater gait variability, and reduced stability [82, 83]. These changes may reflect biomechanical adaptations to excess body weight (e.g., maintaining balance), increased metabolic demand (e.g., higher energy cost), and physiological factors such as muscle weakness [82, 83, 84]. Chronic inflammation and brain atrophy, mechanisms implicated in obesity's effects on AD, may also contribute to gait impairment.

Furthermore, cardiovascular disease, physical inactivity, and metabolic disorders (e.g., hyperglycemia, hyperinsulinemia, insulin resistance: Supplementary File 2) are closely intertwined with obesity. Because obesity can be a cause and consequence of these conditions, they may collectively exacerbate gait impairment and cognitive decline.

#### 3.1.3 Smoking

Epidemiological studies consistently show that former and current smoking increases the risk of dementia [84]. Smoking‐induced cerebral oxidative stress and inflammation are considered key mechanisms underlying neurobiological abnormalities, including Aβ and tau accumulation, as well as structural brain changes [84, 85, 86]. A case–control study comparing smokers and non‐smokers found that active smokers exhibit higher Aβ42 levels, excessive oxidative stress, increased neuroinflammation, and impaired neuroprotection [87].

Smoking also accelerates muscle loss (i.e., sarcopenia) in older adults through multiple physiological pathways [88]. These include oxygen deprivation in muscle tissue, enhanced proteolysis with reduced protein synthesis, and increased susceptibility to muscle damage. Additionally, smoking contributes to peripheral artery disease and systemic inflammation [89]. These effects directly impair gait performance, and reduced physical activity and comorbid respiratory and cardiovascular diseases further compound the impact.

#### 3.1.4 Physical Inactivity

Physical inactivity reduces cerebral perfusion, thereby impairing Aβ clearance, vascular integrity, and neurotrophic support (e.g., brain‐derived neurotrophic factor). These alterations impede neurogenesis and synaptic plasticity [90, 91]. Concurrently, physical inactivity increases oxidative stress and chronic inflammation, contributing to hippocampal atrophy, and cognitive decline [90, 91]. Animal models have provided mechanistic support that treadmill exercise in presenilin‐2 mutant mice attenuates neuronal loss and decreases inflammatory cytokines (tumor necrosis factor‐α, interleukin‐1α), thereby reducing Aβ‐induced neurotoxicity [92]. Consistent findings in humans indicate that physically active individuals exhibit a lower Aβ burden on PiB‐PET and higher cerebrospinal fluid Aβ42 levels [93].

Sedentary behavior is also associated with an increased risk of gait impairment [94]. For example, prolonged daily sitting time is associated with subsequent reductions in gait speed and balance stability [95]. A sedentary lifestyle and gait impairment may mutually reinforce each other, collectively increasing AD risk through overlapping mechanisms, such as hypertension, diabetes, and obesity, thereby reducing blood flow, increasing chronic inflammation, and decreasing brain volume [90, 96]. The temporal directionality of these relationships remains unclear.

#### 3.1.5 Depression

Meta‐analytic evidence indicates that depression confers a two‐ to three‐fold increased risk of dementia [97]. The primary biological mechanisms linking depression to dementia include dysregulation of the hypothalamic–pituitary–adrenal axis with altered glucocorticoid levels, increased Aβ deposition, neuroinflammatory changes, hippocampal atrophy, deficits in neurotrophic factors, and cerebrovascular pathology [98, 99]. Chronic stress associated with depression may further exacerbate these shared neurobiological pathways, heightening the risk of AD.

Psychomotor retardation, a hallmark of depression, manifests as slower gait speed, shorter stride length, and reduced arm swing [100]. Longitudinal data suggest that worsening depressive symptoms correlate with progressive gait slowing [101]. However, the temporal direction remains unclear: gait decline could result from depression‐related neural changes, or early AD pathology could underlie depression and motor slowing. Prospective neuroimaging studies integrating both processes are required to clarify this bidirectional relationship.

### 3.2 Preclinical AD Contributes to Gait Decline

Pathological changes associated with brain degeneration in AD commence at least 10–20 years before the onset of clinical dementia [102, 103]. These changes are characterized by the early deposition of Aβ in the precuneus and other cortical regions within the default mode network (DMN), followed by focal cortical hypometabolism, accumulation of tau pathology, hippocampal atrophy, and the emergence of symptomatic cognitive impairment [104, 105]. As previously mentioned, emerging evidence suggests that these early AD‐related changes may also contribute to impaired gait performance, even before noticeable physical decline (see Section 2.1). These findings suggest a possible mechanism whereby early AD‐related pathological changes may contribute to gait impairment. However, factors driving AD pathology (e.g., cardiovascular condition) may act either prior to or concurrently with Aβ amyloid and tau deposition and accumulation.

Aβ deposition, considered one of the earliest pathological events in AD, may impair gait by damaging brain regions critical for motor coordination and cognition, triggering chronic inflammation, and compromising cerebral vasculature [104, 105]. Although animal studies have linked Aβ accumulation to motor deficits, findings remain inconsistent [106, 107]. One mechanistic study reported that Aβ may impair dopaminergic motor function through neuronal cell loss [108].

Some studies have found that tau pathology, rather than Aβ, is more strongly associated with reduced gait performance [12, 49, 50]. This is plausible given that tauopathy is linked to movement disorders, neuroinflammation, neuronal death, and degeneration of dopamine‐producing neurons [109, 110]. Aβ and tau may influence gait either synergistically or through independent pathways. Given the sequential progression of AD pathology, the observed association between medial temporal atrophy, specifically in the hippocampus, and slower gait may reflect downstream effects of Aβ and tau accumulation.

**FIGURE 3:**
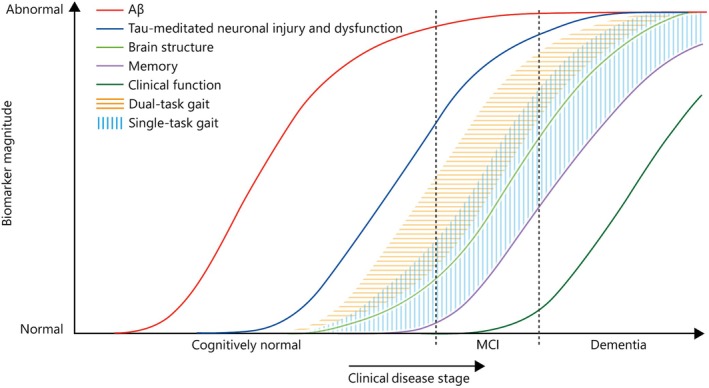
Proposed model of subtle gait impairments, including dual‐task deficits, in the clinical stage of dementia (modified from Jack et al.) [111]. The shaded areas illustrate the hypothesized temporal relationship between Alzheimer's disease‐related pathological changes and gait impairment. In this model, gait impairment, particularly under dual‐task conditions, may emerge after amyloid‐β and tau accumulation but before overt cognitive decline. Although epidemiological studies suggest that gait decline may precede memory impairment, direct evidence on their temporal sequence remains limited, and gait decline, particularly under single‐task conditions, may also occur concurrently with cognitive decline. This model should therefore be interpreted as a conceptual framework rather than a definitive sequence [112]. *Source:* Permission to reproduce has been granted by Elsevier.

**FIGURE 4:**
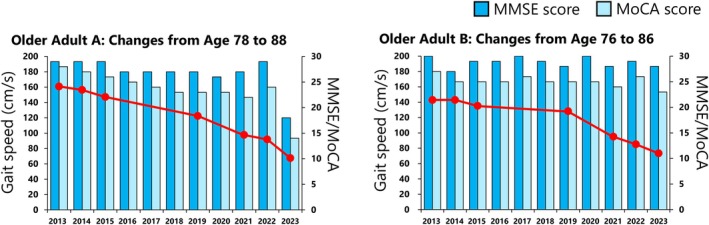
Examples of gait and cognitive changes over 10 years and amyloid‐β accumulation among old–old adults. The data are from an ongoing imaging study, although several gait assessments (2016, 2017, 2018, and 2020) were incomplete [23, 37]. The line chart depicts changes in gait speed, whereas each bar represents scores on cognitive assessments. Older adult A first experienced a decline in gait speed, followed by cognitive decline. This individual was found to be amyloid‐positive on amyloid positron emission tomography (PET) in 2023. Older adult B also experienced a gradual decline in gait speed over the years, similar to A, but no significant cognitive decline was observed. B also underwent amyloid PET in 2023; however, the result was negative. These examples indicate that older adults showing gait speed decline with aging do not always exhibit amyloid deposition and following cognitive impairment based on amyloid cascade hypothesis, and an importance of considering a phenotype in the construction of the association between gait impairment and the emerging Alzheimer's disease pathology.

These findings support the proposal that subtle gait impairments may represent early functional changes along the trajectory toward dementia, potentially emerging before overt cognitive impairment becomes clinically apparent [111] (Figure 3). In particular, dual‐task gait impairment may reflect early disruption of cognitive–motor integration, whereas single‐task gait impairment may capture a broader, less specific motor decline. This phenomenon, in which changes in gait performance precede cognitive decline, is also often observed in cohort studies [113] (Figure 4). However, the stage at which gait impairment emerges remains uncertain, as no systematic studies have addressed this question. As noted in the prior research agenda [112], variability in clinical conditions, lifestyle factors, and residual physical ability may contribute to individual differences in the timing of gait impairment associated with AD pathology, particularly in simple‐task gait.

In addition to these direct neurobiological pathways, reduced physical activity that emerges during the preclinical phase of AD may also contribute indirectly to gait decline. A recent cohort study with a 27‐year follow‐up suggested that the observed association between lower physical activity levels and an increased risk of dementia may be partly explained by reverse causation; that is, declining activity levels may already reflect the preclinical stage of dementia [114]. In this context, reduced physical activity may not only be an early behavioral manifestation of preclinical dementia but may also contribute to subsequent gait impairment through long‐term disuse.

### 3.3 Persistent Gait Impairment May Aggravate AD Pathology

If gait performance declines before cognitive impairment and the onset of AD pathology, several underlying contributors may be involved. These include asymptomatic cerebrovascular disease, neuromuscular disorders, orthopedic conditions, and age‐related disuse syndrome (i.e., physical inactivity). In this context, reduced mobility and disease burden resulting from impaired gait may act as mediators between gait dysfunction and AD pathology. This proposed mechanism remains hypothetical and should be interpreted with caution. Gait decline alone is unlikely to directly cause cognitive impairment or initiate AD‐related pathology but may interact with pre‐existing brain vulnerability, systemic risk factors, and compensatory neural processes.

Even though gait impairment is thought to be a phenomenon that often occurs in parallel with AD pathology accumulation, recent research also suggests that persistent impaired gait function may accelerate AD‐related neurodegeneration [115]. Functional neuroimaging studies indicate that older adults exhibit increased brain activation during actual or imagined walking, compared with younger individuals, potentially reflecting compensatory neural recruitment [116]. For example, during imagined adaptive locomotion, older adults demonstrate greater activation in the bilateral presupplementary motor area, dorsal and ventral premotor cortices, precentral gyrus, posterior parietal lobes, and visual association areas, compared with younger adults [117]. Similarly, a study using functional near‐infrared spectroscopy has demonstrated that older adults exhibit elevated prefrontal activity during gait tasks, relative to younger participants [118]. Such findings align with the concept that aging and peripheral impairments, such as muscle weakness, sensory loss, and balance deficits, disrupt the automatic processing of sensory and motor information necessary for gait control. To compensate, higher‐order motor regions, such as the prefrontal cortex, become increasingly engaged in tasks that are typically automatic. This compensatory “overinvolvement” elevates neural activity in regions that overlap with hubs of the DMN.

Human studies have revealed that heightened activity in network hubs (i.e., DMN) often co‐localizes with tau deposition in AD [119, 120], whereas animal studies suggest a direct link between excessive neuronal activity and AD pathology [121]. Therefore, chronic and uncontrolled overactivation of DMN‐related areas, including the prefrontal, cingulate, medial temporal, and posterior parietal cortices due to gait impairment, could potentially contribute to or exacerbate AD‐related pathological processes [115, 119]. This hypothesis is further supported by findings that lower resting‐state activity in DMN regions, commonly affected in AD, is associated with poorer gait performance in older adults [23, 37]. Although this challenging mechanism has previously been proposed to explain how hearing loss may contribute to dementia (see Supplementary File 2), it remains speculative and has not been directly validated in human studies.

### 3.4 Life‐Course Origins

A longitudinal study spanning five decades revealed that individuals exhibiting slower gait speed at the age of 45 years had shown lower intelligence quotient (IQ) scores in childhood (ages 7–11 years), as well as a more pronounced rate of cognitive decline by midlife [122]. This association between midlife gait speed and early‐life cognitive function was also reflected in a composite brain health score, encompassing measures, such as picture vocabulary, receptive language, motor skills, and behavioral control. This suggests that the link between gait and cognitive performance in midlife and older age may stem from disruptions in brain development beginning in early life, supporting a neurodevelopmental origin model [123]. This hypothesis is plausible given that childhood represents a critical period of neural development, characterized by the rapid maturation of sensory and motor systems. During this time, the brain is particularly receptive to environmental stimuli, which play a crucial role in shaping the neural circuits underlying perception and motor function. Indeed, lower educational attainment is a risk factor for dementia and gait impairment (Figure 2) [58, 61].

These results are consistent with previous birth‐cohort studies indicating that higher childhood IQ is associated with better lung function in later life [124]. Together, slow gait in middle age and beyond may reflect “accumulated risk from developmental stages.” In essence, individuals with greater neurocognitive and sensorimotor resources in early life may engage in more physical activity, maintain better gait performance, and exhibit increased resilience to aging [125]. Conversely, limited early‐life resources may lead to poorer gait performance and potentially accelerate age‐related decline [125]. Such life‐course influences may underlie the observed relationship between gait and cognitive performance, shaping susceptibility and resilience to AD‐related and mixed neurodegenerative processes. This hypothesis is partially supported by previous findings indicating that early life adversities, stressful or traumatic events occurring before the age of 18 years, can disrupt the maturation of neural and physiological systems [125], impair immune function accelerating immunosenescence [126], and increase the risk of falls in old age [127], and in severe cases, elevate the risk of mortality [128]. Although causality remains to be established, accumulating evidence suggests that early‐life experiences may play a critical role in shaping individual susceptibility and resilience to gait decline and cognitive impairment in later life. This life‐course perspective underscores the need to move beyond domain‐specific approaches and to consider gait and cognition as integrated outcomes of lifelong brain–body health.

## 4 Future Directions for Healthy Gait and Cognition

Human physiology integrates an intricate complex network of control systems and feedback loops that enables the human body to perform a variety of functions necessary for survival. With aging, two of our more sophisticated and complex human systems: cognition and gait, start to deteriorate and can lead to the functional decline associated with severe mobility impairment and dementia. This review supports the concept that gait performance is a plausible motor marker of vulnerability to AD‐related and mixed brain pathology. Although some conflicting findings have been noted, as discussed in Section 2, we hypothesize that the association between gait impairment and AD‐related pathology may be mediated by several mechanisms. These mechanisms are not mutually exclusive and are likely to overlap or interact dynamically, highlighting the importance of assessing lifestyle and health factors that influence gait performance and AD pathology not only in old age but also earlier in life. Therefore, it is imperative for health professionals to adopt a comprehensive, life‐course perspective on health rather than focusing solely on a single functional decline in later life. Furthermore, such lifelong interactions among risk factors may help explain the variability in findings regarding the association between gait disturbances and emerging AD pathology or AD onset. Indeed, when examining the progression of functional changes among community‐dwelling older adults, gait performance often declines; however, some individuals maintain cognitive function and do not develop AD (Figure 4). Further investigation into the mechanisms underlying this phenotype will deepen our understanding of gait performance as a motor marker that reflects, rather than drives, AD pathology and will help distinguish pathological changes from normal age‐related gait decline.

During the last decade, up to nine additional pathological changes have been identified in association with clinical AD, including microvascular changes, white matter hyperintensities, neuroinflammation, gliosis, and microhemorrhages, supporting the concept of mixed pathology for AD [21]. This review focuses on the ATN classification to examine the association between AD pathology and gait impairment and, therefore, did not address mixed pathologies in detail. Although individual changes, such as white matter hyperintensities, have been shown to be closely linked to impaired gait performance [129], clarifying the temporal dynamics and phenotypic patterns of these associations will help determine what age‐related gait decline signifies in the context of pathological aging, ultimately contributing to a deeper understanding of the physiological complexity of human aging.

Individuals experiencing concurrent declines in gait speed and cognition are at the highest risk of progression to dementia [68, 130]. Considering the diverse and complex underlying mechanisms, it is important to adopt a holistic approach to maintain health in old age, rather than focusing on a single functional decline or unhealthy lifestyle. This is consistent with recent concepts on dementia prevention. For example, following the famous FINGER Trial [131], the J‐MINT study, a multifactorial intervention conducted in Japan, demonstrated improvements in cognitive function among older adults with MCI, particularly in *APOE* ε4 carriers [132]. Similarly, the SYNERGIC trial in Canada, which combined interventions of exercise, cognitive activity, and vitamin D, demonstrated improvements in cognition and gait performance, as well as a reduction in falls rate at 12 months of intervention, suggesting that the combined therapy had a synergistic effect when compared with physical exercises alone [133, 134]. Such a holistic strategy should be adopted at each stage to prevent the association of declining gait performance with emerging AD pathology.

Recent research has increasingly categorized declines in specific domains, such as “cognitive frailty,” “mental frailty,” “oral frailty,” and “social frailty,” as independent conditions. Although this trend highlights domain‐specific vulnerabilities, it risks fragmenting our understanding of the aging process and may lead to misinterpretation of the functional components of older adults as independent rather than interconnected [135]. In the context of gait and cognition, such compartmentalized views may obscure the fact that declines in physical and cognitive functions often arise from shared mechanisms. From this perspective, addressing gait slowing or cognitive decline in isolation may fail to capture their interdependence. Instead, a comprehensive, life‐course approach is required to shape individual susceptibility and resilience to gait decline and cognitive impairment. Rather than viewing gait or cognition as separate entities, they should be understood as interlinked expressions of overall brain–body health, requiring coordinated preventive strategies long before advanced age.

## Disclosure

The authors have nothing to report.

## Ethics Statement

The authors have nothing to report.

## Supporting information


**Supplementary 1:** Search strategy and study selection.

**Supplementary 2:** Additional potentially shared modifiable risk factors.

## Data Availability

Data sharing not applicable to this article as no datasets were generated or analysed during the current study.
