# Supplementary material for: Gait Impairment and Alzheimer's Disease Pathology: A Narrative Review on Mechanistic Links
Source: Geriatr Gerontol Int. 2026 Aug 2;26(8):e70675. doi: 10.1111/ggi.70675 (PMC13429374; doi:10.1111/ggi.70675)
Supplement: Supplementary file 1 — Supplementary 1: Search strategy and study selection. [file GGI-26-0-s001.docx]

## Search strategy and study selection

Database searches were conducted in PubMed and ScienceDirect in September 2025 using the following search strategy: (elderly OR older adults) AND (gait OR walking) AND (amyloid OR tau OR hippocampus OR brain atrophy). Articles published in English between 2000 and 2025 were eligible for inclusion. Additional grey literature was identified through Google and Google Scholar, and relevant citations and reference lists were hand-searched. Search limiters included publication year (2000–2025), language (English), human participants, peer-reviewed journal articles, and observational study designs (quantitative, longitudinal, follow-up, prospective, and retrospective).

Titles and abstracts were independently screened for relevance. Studies were then classified according to participant group (cognitively healthy older adults or individuals with mild cognitive impairment) and gait assessment type (single-task or dual-task walking). Full-text articles meeting these criteria were reviewed, and findings were selected based on their alignment with the objectives of this review.

Because this was a narrative review, no formal risk-of-bias assessment was conducted. However, studies were evaluated descriptively with attention to study design, participant characteristics, gait assessment methods, biomarker or neuroimaging measures, and consistency of reported findings.
