# Supplementary material for: Gait Impairment and Alzheimer's Disease Pathology: A Narrative Review on Mechanistic Links
Source: Geriatr Gerontol Int. 2026 Aug 2;26(8):e70675. doi: 10.1111/ggi.70675 (PMC13429374; doi:10.1111/ggi.70675)
Supplement: Supplementary file 2 — Supplementary 2: Additional potentially shared modifiable risk factors. [file GGI-26-0-s002.docx]

# **Additional potentially shared modifiable risk factors**

## **Metabolic disorders**

Metabolic disorders, particularly insulin resistance, may promote Aβ deposition through mechanisms that affect both the production and clearance of Aβ in the brain.^1^ In response to insulin resistance, the body compensates by producing excess insulin to maintain normal blood glucose levels. This surplus insulin can cross the blood–brain barrier and is degraded by insulin-degrading enzyme (IDE), which also targets Aβ. During hyperinsulinemia, insulin competes with Aβ for IDE-mediated degradation, thereby reducing Aβ clearance and promoting its accumulation.^2^ Furthermore, hyperglycemia and oxidative stress may exacerbate neuroinflammation, vascular damage, and cerebral hypoperfusion, all of which contribute to neuronal injury.^1-3^ T Type 2 diabetes mellitus may also increase the risk of AD through central insulin resistance, impaired glucose metabolism, and enhanced Aβ and tau aggregation.^4, 5^

Individuals with diabetes frequently exhibit slower, less stable gait patterns.^6, 7^ Peripheral neuropathy, sensory deficits, reduced lower-extremity strength, and central nervous system alterations, including reduced white matter integrity, may jointly contribute to gait dysfunction in diabetes.^8^

## **Visual loss**

Visual loss may be both a consequence of and a contributing factor to Aβ accumulation and related neurodegeneration. The relationship is complex and may involve bidirectional pathways between the eye and the brain.^9, 10^ Several mechanisms have been proposed to explain how visual loss may influence AD onset.^9^ First, visual impairment and AD may share common risk factors and disease spectra that contribute to cognitive decline.^9^ Second, reduced social engagement among individuals with visual impairment may lead to diminished cognitive stimulation and higher rates of depression.^11^ Third, greater effort is required to perceive and interpret sensory information in the presence of visual impairment, increasing cognitive load and potentially elevating dementia risk.^12^

Visual loss is also linked to gait impairment in older adults, as vision is critical for movement planning, balance, and hazard detection.^13-15^ Impaired vision often results in a cautious and less efficient gait pattern, characterized by slower walking speed and increased fall risk, particularly when navigating obstacles or uneven surfaces. This gait impairment may be further exacerbated by disuse syndrome resulting from inactivity associated with visual loss.

## **Hearing loss**

Hearing loss has recently emerged as a potential risk factor for dementia, with several mechanisms proposed to explain this association.^16^ One prominent theory is the diminished auditory stimulation hypothesis, which proposes that reduced sensory input caused by hearing impairment lowers neural activity in auditory and memory-related regions, such as the hippocampus, potentially accelerating cognitive decline. Another explanation is the cognitive overload hypothesis, which posits that the increased mental effort and compensatory overuse of the medial temporal region required to comprehend speech in individuals with hearing loss may contribute to Aβ and tau accumulation in this region.^16^ While these theories appear plausible, conclusive evidence is lacking.

Previous studies have shown that older adults with hearing impairment exhibit slower gait speed and increased stride variability.^17-19^ This phenomenon may be partly attributable to the loss of auditory input as a sensory anchor, similar to visual fixation or light touch, which helps individuals perceive their surroundings and maintain balance during locomotion.^20, 21^ Physical inactivity may partially mediate this association, as hearing loss can contribute to a more sedentary lifestyle.^22^ The extent to which gait impairment associated with hearing loss is influenced by AD pathology, as previously described, remains unclear.

## **Traumatic brain injury**

An epidemiological link between traumatic brain injury (TBI) and the later development of AD is well established.^23-25^ Evidence suggests that Aβ deposition can be detected within hours after TBI.^26^ In the acute phase, axonal damage and accumulation of amyloid precursor protein may lead to rapid Aβ aggregation.^26, 27^ Over the long term, TBI may be associated with sustained increases in Aβ burden through chronic inflammation and impaired clearance, potentially increasing the likelihood of Alzheimer’s-like pathology.^26, 27^

Gait impairment is observed following TBI regardless of injury severity.^28^ Declines in mobility are thought to result from disruption of motor control systems due to axonal damage.^29^ Furthermore, cerebral microbleeds associated with TBI have been implicated in long‑term gait disturbances among older adults.^30^

### **Non-modifiable factors in shared risks**

#### Age and sex

Several aging-related processes, including increased inflammation, oxidative stress, impaired metabolism, DNA damage, and declining brain maintenance functions such as waste clearance, may create a permissive environment for the abnormal accumulation of Aβ and tau proteins. This accumulation contributes to synaptic dysfunction, neuronal loss, and ultimately an increased risk of AD.^31, 32^ Sex is also associated with AD risk, with women having a greater lifetime likelihood of developing the disease.^33, 34^ Although the underlying mechanisms remain unclear, sex‑related differences in hormonal changes (particularly the decline in estrogen during menopause), life experiences, cardiovascular risks (e.g., hypertension during pregnancy), neuroinflammation, epigenetics, metabolism, autophagy, and potential genetic factors may all contribute to elevated AD risk.^34, 35^

Aging is also closely linked to impaired gait performance. Several physiological factors are involved, with declining neuromuscular function providing one of the most direct explanations.^36^ Such age-related neuromuscular changes may be partly driven by comorbidities and physical inactivity. Moreover, women typically exhibit slower gait speeds than men, largely due to sex-related differences in biomechanics, such as shorter leg length, physiology, such as lower muscle mass, and potentially psychological factors.^37^

#### *APOE* ε4 genotype

*APOE* ε4 is the primary genetic risk factor for late-onset AD and its associated cognitive impairments.^38, 39^ *APOE*4 may increase the risk of AD by promoting the accumulation of Aβ and tau proteins, disrupting lipid metabolism, and promoting neuroinflammation. It may also interfere with normal cholesterol transport, leading to lipid accumulation in brain cells and impaired cellular function, while contributing to synaptic dysfunction and cerebrovascular abnormalities. Indeed, *APOE* ε4 carriage increases the risk of cardiovascular disease.^40^

Emerging evidence links *APOE* ε4 to physical functioning, including gait performance.^41^ A population-based cross-sectional study found that older adults carrying the ε4 allele had slower gait speeds than non-carriers.^42^ Longitudinal data indicate that ε4 carriage predicts future gait decline among older adults with MCI.^43^ A recent study further demonstrated that elevated GFAP, a marker of astroglial activation, mediates the association between *APOE* ε4 carriage and slow gait, suggesting the involvement of neuroinflammation. Given findings that *APOE* ε4 carriers without gait impairment may be less likely to experience cognitive decline,^44^ certain behavioral or physiological factors may contribute to elevated GFAP expression, which in turn may impair both gait and cognitive function. However, because not all ε4 carriers develop dementia,^39, 45^ its contribution to the gait–AD linkage should be interpreted as probabilistic rather than deterministic.

# **References**

[1] Yue C, Fu Y, Zhao Y, Ou Y, Sun Y, Tan L. Association between Alzheimer’s disease and metabolic syndrome: Unveiling the role of dyslipidemia mechanisms. *Brain Network Disorders* 2025; **1**: 21-27. doi: <https://doi.org/10.1016/j.bnd.2024.10.006>

[2] Bosco D, Fava A, Plastino M, Montalcini T, Pujia A. Possible implications of insulin resistance and glucose metabolism in Alzheimer's disease pathogenesis. *J Cell Mol Med* 2011; **15**: 1807-21. doi: 10.1111/j.1582-4934.2011.01318.x

[3] Raza A, Saleem S, Imran S, et al. From metabolic dysregulation to neurodegenerative pathology: the role of hyperglycemia, oxidative stress, and blood-brain barrier breakdown in T2D-driven Alzheimer's disease. *Metab Brain Dis* 2025; **40**: 276. doi: 10.1007/s11011-025-01700-z

[4] Naing HL, Teo SP. Impact of Hypertension on Cognitive Decline and Dementia. *Ann Geriatr Med Res* 2020; **24**: 15-19. doi: 10.4235/agmr.19.0048

[5] Stanciu GD, Bild V, Ababei DC, et al. Link Between Diabetes and Alzheimer's Disease due to the Shared Amyloid Aggregation and Deposition Involving both Neurodegenerative Changes and Neurovascular Damages. *J Clin Med* 2020; **9**doi: 10.3390/jcm9061713

[6] Han F, Kong X, Lv W, Li S, Sun Y, Wu Y. Association of diabetes mellitus with gait and falls in community-dwelling older adults: Serial mediation of vision and cognition. *Archives of Gerontology and Geriatrics* 2023; **104**: 104827. doi: <https://doi.org/10.1016/j.archger.2022.104827>

[7] Maksimovic A, Hanewinckel R, Verlinden VJA, et al. Gait characteristics in older adults with diabetes and impaired fasting glucose: The Rotterdam Study. *Journal of Diabetes and its Complications* 2016; **30**: 61-66. doi: <https://doi.org/10.1016/j.jdiacomp.2015.10.006>

[8] Alam U, Riley DR, Jugdey RS, et al. Diabetic Neuropathy and Gait: A Review. *Diabetes Ther* 2017; **8**: 1253-64. doi: 10.1007/s13300-017-0295-y

[9] Zheng C, Zeng R, Wu G, Hu Y, Yu H. Beyond Vision: A View from Eye to Alzheimer's Disease and Dementia. *The Journal of Prevention of Alzheimer's Disease* 2024; **11**: 469-83. doi: <https://doi.org/10.14283/jpad.2023.118>

[10] Wang L, Mao X. Role of Retinal Amyloid-β in Neurodegenerative Diseases: Overlapping Mechanisms and Emerging Clinical Applications. *Int J Mol Sci* 2021; **22**doi: 10.3390/ijms22052360

[11] Hong T, Mitchell P, Burlutsky G, Gopinath B, Liew G, Wang JJ. Visual impairment and depressive symptoms in an older Australian cohort: longitudinal findings from the Blue Mountains Eye Study. *British Journal of Ophthalmology* 2015; **99**: 1017-21. doi: 10.1136/bjophthalmol-2014-306308

[12] Chen SP, Azad AD, Pershing S. Bidirectional Association between Visual Impairment and Dementia Among Older Adults in the United States Over Time. *Ophthalmology* 2021; **128**: 1276-83. doi: <https://doi.org/10.1016/j.ophtha.2021.02.021>

[13] West SK, Rubin GS, Broman AT, Muñoz B, Bandeen-Roche K, Turano K. How does visual impairment affect performance on tasks of everyday life? The SEE Project. Salisbury Eye Evaluation. *Arch Ophthalmol* 2002; **120**: 774-80. doi: 10.1001/archopht.120.6.774

[14] Salive ME, Guralnik J, Glynn RJ, Christen W, Wallace RB, Ostfeld AM. Association of visual impairment with mobility and physical function. *J Am Geriatr Soc* 1994; **42**: 287-92. doi: 10.1111/j.1532-5415.1994.tb01753.x

[15] Swenor BK, Simonsick EM, Ferrucci L, Newman AB, Rubin S, Wilson V. Visual impairment and incident mobility limitations: the health, aging and body composition study. *J Am Geriatr Soc* 2015; **63**: 46-54. doi: 10.1111/jgs.13183

[16] Griffiths TD, Lad M, Kumar S, et al. How Can Hearing Loss Cause Dementia? *Neuron* 2020; **108**: 401-12. doi: 10.1016/j.neuron.2020.08.003

[17] Li L, Simonsick EM, Ferrucci L, Lin FR. Hearing loss and gait speed among older adults in the United States. *Gait Posture* 2013; **38**: 25-9. doi: 10.1016/j.gaitpost.2012.10.006

[18] Sakurai R, Kawai H, Yanai S, et al. Gait and Age-Related Hearing Loss Interactions on Global Cognition and Falls. *Laryngoscope* 2022; **132**: 857-63. doi: 10.1002/lary.29898

[19] Sakurai R, Suzuki H, Ogawa S, Takahashi M, Fujiwara Y. Hearing loss and increased gait variability among older adults. *Gait Posture* 2021; **87**: 54-58. doi: 10.1016/j.gaitpost.2021.04.007

[20] Campos J, Ramkhalawansingh R, Pichora-Fuller MK. Hearing, self-motion perception, mobility, and aging. *Hear Res* 2018; **369**: 42-55. doi: 10.1016/j.heares.2018.03.025

[21] Carpenter MG, Campos JL. The Effects of Hearing Loss on Balance: A Critical Review. *Ear Hear* 2020; **41 Suppl 1**: 107s-19s. doi: 10.1097/aud.0000000000000929

[22] Kuo P-L, Di J, Ferrucci L, Lin FR. Analysis of Hearing Loss and Physical Activity Among US Adults Aged 60-69 Years. *JAMA Network Open* 2021; **4**: e215484-e84. doi: 10.1001/jamanetworkopen.2021.5484

[23] Mortimer JA, van Duijn CM, Chandra V, et al. Head trauma as a risk factor for Alzheimer's disease: a collaborative re-analysis of case-control studies. EURODEM Risk Factors Research Group. *Int J Epidemiol* 1991; **20 Suppl 2**: S28-35. doi: 10.1093/ije/20.supplement_2.s28

[24] Graves AB, White E, Koepsell TD, et al. The association between head trauma and Alzheimer's disease. *Am J Epidemiol* 1990; **131**: 491-501. doi: 10.1093/oxfordjournals.aje.a115523

[25] Livingston G, Huntley J, Liu KY, et al. Dementia prevention, intervention, and care: 2024 report of the <em>Lancet</em> standing Commission. *The Lancet* 2024; **404**: 572-628. doi: 10.1016/S0140-6736(24)01296-0

[26] Johnson VE, Stewart W, Smith DH. Traumatic brain injury and amyloid-β pathology: a link to Alzheimer's disease? *Nat Rev Neurosci* 2010; **11**: 361-70. doi: 10.1038/nrn2808

[27] Washington PM, Morffy N, Parsadanian M, Zapple DN, Burns MP. Experimental traumatic brain injury induces rapid aggregation and oligomerization of amyloid-beta in an Alzheimer's disease mouse model. *J Neurotrauma* 2014; **31**: 125-34. doi: 10.1089/neu.2013.3017

[28] Dever A, Powell D, Graham L, et al. Gait Impairment in Traumatic Brain Injury: A Systematic Review. *Sensors (Basel)* 2022; **22**doi: 10.3390/s22041480

[29] Merchant RA, Wojszel ZB, Ryg J. The hidden impact of mild-traumatic brain injury in older adults. *European Geriatric Medicine* 2024; **15**: 1551-54. doi: 10.1007/s41999-024-01075-x

[30] Toth L, Czigler A, Horvath P, et al. Traumatic brain injury-induced cerebral microbleeds in the elderly. *Geroscience* 2021; **43**: 125-36. doi: 10.1007/s11357-020-00280-3

[31] Liu Y, Tan Y, Zhang Z, Yi M, Zhu L, Peng W. The interaction between ageing and Alzheimer's disease: insights from the hallmarks of ageing. *Transl Neurodegener* 2024; **13**: 7. doi: 10.1186/s40035-024-00397-x

[32] Alrouji M, Alshammari MS, Tasqeeruddin S, Shamsi A. Interplay Between Aging and Tau Pathology in Alzheimer's Disease: Mechanisms and Translational Perspectives. *Antioxidants (Basel)* 2025; **14**doi: 10.3390/antiox14070774

[33] Rajan KB, Weuve J, Barnes LL, McAninch EA, Wilson RS, Evans DA. Population estimate of people with clinical Alzheimer's disease and mild cognitive impairment in the United States (2020–2060). *Alzheimer's & Dementia* 2021; **17**: 1966-75. doi: <https://doi.org/10.1002/alz.12362>

[34] Lopez-Lee C, Torres ERS, Carling G, Gan L. Mechanisms of sex differences in Alzheimer’s disease. *Neuron* 2024; **112**: 1208-21. doi: <https://doi.org/10.1016/j.neuron.2024.01.024>

[35] Pike CJ. Sex and the development of Alzheimer's disease. *J Neurosci Res* 2017; **95**: 671-80. doi: 10.1002/jnr.23827

[36] Song S, Geyer H. Predictive neuromechanical simulations indicate why walking performance declines with ageing. *J Physiol* 2018; **596**: 1199-210. doi: 10.1113/jp275166

[37] Ko SU, Tolea MI, Hausdorff JM, Ferrucci L. Sex-specific differences in gait patterns of healthy older adults: results from the Baltimore Longitudinal Study of Aging. *J Biomech* 2011; **44**: 1974-9. doi: 10.1016/j.jbiomech.2011.05.005

[38] Cosentino S, Scarmeas N, Helzner E, et al. APOE epsilon 4 allele predicts faster cognitive decline in mild Alzheimer disease. *Neurology* 2008; **70**: 1842-9. doi: 10.1212/01.wnl.0000304038.37421.cc

[39] Farrer LA, Cupples LA, Haines JL, et al. Effects of age, sex, and ethnicity on the association between apolipoprotein E genotype and Alzheimer disease. A meta-analysis. APOE and Alzheimer Disease Meta Analysis Consortium. *Jama* 1997; **278**: 1349-56.

[40] Saeed A, Lopez O, Cohen A, Reis SE. Cardiovascular Disease and Alzheimer's Disease: The Heart&#x2013;Brain Axis. *Journal of the American Heart Association* 2023; **12**: e030780. doi: doi:10.1161/JAHA.123.030780

[41] Verghese J, Holtzer R, Wang C, Katz MJ, Barzilai N, Lipton RB. Role of APOE genotype in gait decline and disability in aging. *J Gerontol A Biol Sci Med Sci* 2013; **68**: 1395-401. doi: 10.1093/gerona/glt115

[42] Doi T, Shimada H, Makizako H, Tsutsumimoto K, Uemura K, Suzuki T. Apolipoprotein E genotype and physical function among older people with mild cognitive impairment. *Geriatr Gerontol Int* 2015; **15**: 422-7. doi: 10.1111/ggi.12291

[43] Sakurai R, Montero-Odasso M. Apolipoprotein E4 Allele and Gait Performance in Mild Cognitive Impairment: Results From the Gait and Brain Study. *J Gerontol A Biol Sci Med Sci* 2017; **72**: 1676-82. doi: 10.1093/gerona/glx075

[44] Sakurai R, Watanabe Y, Osuka Y, et al. Overlap Between Apolipoprotein Eε4 Allele and Slowing Gait Results in Cognitive Impairment. *Front Aging Neurosci* 2019; **11**: 247. doi: 10.3389/fnagi.2019.00247

[45] Ferrari C, Xu WL, Wang HX, et al. How can elderly apolipoprotein E ε4 carriers remain free from dementia? *Neurobiol Aging* 2013; **34**: 13-21. doi: 10.1016/j.neurobiolaging.2012.03.003
